# Long-term prosthesis survival of total hip and total knee arthroplasty in people with inherited bleeding disorders

**DOI:** 10.1016/j.rpth.2026.106887

**Published:** 2026-07-28

**Authors:** Gijs Aertssen, Huub M. de Visser, Wouter Foppen, Roger E.G. Schutgens, Merel A. Timmer, Lize F.D. van Vulpen

**Affiliations:** 1 Center for Benign Haematology, Thrombosis and Haemostasis, Van Creveldkliniek, University Medical Center Utrecht, Utrecht University, Utrecht, The Netherlands; 2 Department of Orthopaedics, Division of Surgical Specialties, University Medical Center Utrecht, Utrecht University, Utrecht, The Netherlands; 3 Department of Orthopaedics, Sint Antonius Hospital, Utrecht, The Netherlands; 4 Department of Radiology and Nuclear Medicine, Division of Imaging & Oncology, University Medical Center Utrecht, Utrecht University, Utrecht, The Netherlands

**Keywords:** haemophilia, joint replacement, survival, total hip, total knee

## Abstract

**Background:**

Joint arthroplasty provides pain relief and functional improvement, but higher peri- and postoperative risks in people with bleeding disorders historically lead to lower prosthesis survival rates (PSRs) than in the general population. Long-term outcomes after advances in hemophilia care remain unclear. In people with bleeding disorders, PSR is 84.0% at 15 years after total knee arthroplasty (TKA) and 91.9% at 5 years after total hip arthroplasty (THA), compared with 92.8% and 93.7% in the general population.

**Objectives:**

To determine PSRs of TKA and THA up to 25 years after surgery in people with end-stage hemophilic arthropathy and to identify predictors for prosthesis failure.

**Methods:**

This single-center study analyzed routine-care data of people with bleeding disorders (1989-2025), with first revision as the endpoint. PSRs and predictors were assessed using Kaplan–Meier and Cox proportional hazards models. Complete follow-up data on pre-, peri- and postoperative characteristics were evaluated; all variables with a univariable *P* < .1 were included in multivariable models.

**Results:**

A total of 128 TKAs and 64 THAs were included. The PSR for TKA in people with bleeding disorders was 92.2% (number at Risk [nR] = 71; 95% CI, 87.5-97.3) after 15 years and 90.9% (nR = 28; 95% CI, 85.5-96.6) after 25 years. The PSR for THA in people with bleeding disorders was 91.0% (nR = 27; 95% CI, 82.8-100) after 15 years and 78.6% (nR = 14; 95% CI, 64.8-95.5) after 25 years. Prosthetic joint infection, more common in patients with an inhibitor, predicted TKA failure. A cemented femoral stem, associated with a higher age, predicted THA failure.

**Conclusion:**

The prothesis survival rate of TKA and THA for people with bleeding disorders with end-stage hemophilic arthropathy was comparable to that of the general population in the literature.

## Essentials

• It is still unclear how long joint replacements last in people with bleeding disorders.
• We included all hip and knee replacements in people with bleeding disorders followed at Van Creveldkliniek since 1989.
• This study reports prosthesis survival up to 25 years and identifies predictors of failure.
• Long-term hip and knee prosthesis survival of people with bleeding disorders matches that of the general population.

## 1 Introduction

Hemophilic arthropathy (HA) causes pain and limited range of motion, leading to limitations in daily activities [1]. The incidence of HA is decreasing, in line with a reduction of joint bleeding episodes from 25 bleeds per year per patient in 1971 to 2 bleeds in 2019 [2]. However, HA is still highly prevalent among patients with bleeding disorders, and emerging evidence suggests that subclinical bleeding also contributes to the development of HA [[3], [4], [5]]. Currently, one-fifth of the adolescents in the Netherlands with factor VIII (FVIII) activity <5% already show abnormalities on X-rays in one or more joints, increasing to almost all patients in their 40s [6,7].

Treatment of HA aims to relieve the symptoms and maintain physical functioning, primarily through nonsurgical interventions like physiotherapy and pain management. When pain and functional limitations persist, total joint replacement (TJR) is considered. However, the timing and indication of surgery differ from those of the general population. HA is already highly prevalent at a relatively young age, involves multiple joints, and results in severe joint deformities, synovial changes, and neovascularization, which complicate surgery and rehabilitation. Therefore, both clinicians and patients must constantly weigh the long-term benefits of arthroplasty against the burden of revision surgery and the high potential for complications [8]. Specifically, prosthetic joint infections (PJIs), aseptic loosening of the joint, and postoperative bleeding are prevalent after TJR in people with bleeding disorders [8,9], as they are associated with (subclinical) bleeding [10,11]. Consequently, prosthesis survival rates (PSRs) were historically worse for people with bleeding disorders than for the general population. Several studies have reported these PSRs in people with bleeding disorders, with follow-up periods extending up to 15 years for total knee arthroplasty (TKA), but only up to 5 years for total hip arthroplasty (THA) [5,8,[12], [13], [14], [15], [16], [17], [18], [19], [20], [21], [22], [23], [24]]. For TKA, the 15-year PSR was 84% in a European cohort [8]; for THA, the 5-year PSR was 91.9% in an American cohort [22,23]. In comparison, in a study of the Dutch general population (all indications, 86.4% osteoarthritis), patients with TKA and THA had a PSR of respectively 92.8% and 93.7% after 15 years [25,26].

Developments in hemophilia care, such introduction of the prophylactic factor (1970) and nonfactor therapies (2017), preoperative embolization in TKA (2000), diminished HIV and hepatitis C infection rates (mid-1990s) together with improved peri- and postoperative management have led to reduced complication rates over the last 2 decades [14,27,28]. Recent studies report improved survival rates that are approaching those observed in the general population, with a PSR of 94.7% at 15 years for TKA and 95.2% at 10 years for THA [12,13,20,[29], [30], [31], [32], [33], [34]]. However, these findings are based on relatively small cohorts, and PSRs extending past 2 decades are still lacking [27]. Therefore, we aimed to determine 15- and 25-year PSRs and long-term complication rates after TKA or THA in patients with end-stage HA. Furthermore, we aimed to identify predictors for failure of the prosthesis. We hypothesized that developments as preoperative embolization and improved (nonfactor) prophylactic treatment are predictors of prolonged prothesis survival [8], as both decrease bleeding risk. In contrast, we hypothesized that having an inhibitor, HIV/hepatitis C infections, postoperative bleeding, and PJI are related to a higher complication rate [9,10] and thus shorter survival.

## 2 Methods

### 2.1 Study design and population

In this single-center retrospective cohort study, regular-care data were extracted from medical records of people with bleeding disorders who underwent TKA or THA who attended the Van Creveldkliniek at University Medical Center Utrecht between 1989 and 2025. Patients with moderate or severe hemophilia A or B (FVIII/FIX <5%) or von Willebrand disease (VWD) with a FVIII activity <5% were included, as arthropathy in patients with these factor levels is assumed to be bleeding-related [35]. All types of primary THA or TKA were included. All patients were followed according to standard care. Patients could be included multiple times with distinct joints. The study was approved by the institutional Research Quality Coordinator of Division Internal Medicine and Dermatology of the University Medical Center Utrecht (25U-0426). Informed consent was waived because the health care data were anonymized. Patients that objected to the use of their (anonymized) health care data were excluded.

### 2.2 Standard care

The Van Creveldkliniek at UMC Utrecht is a multidisciplinary hemophilia treatment center providing lifelong care, with regular monitoring and treatment of bleeding disorders and close surveillance of joint health through routine visits and imaging. Physiotherapy was used to prevent or treat limitations in physical functioning as a consequence of HA and to rehabilitate after joint bleeds. When persistent pain and functional limitations remain, TJR is indicated. For every surgery, the local hemostatic protocol was used: factor levels were increased to 80% to 100% at day of surgery and at day 1. From days 2 to 7, factor levels are kept at 50%, and from days 7 to 10 (THA) or 14 (TKA), factor levels are kept at 30% either by continuous infusion of coagulation factor or bolus therapy. Because surgeries are complex and expensive in this population, and multiple joints might be affected, procedures performed on different joints might be combined in one admission (multiple joint procedures), such as bilateral procedures or together with an ankle arthrodesis. In uncomplicated, unilateral procedures, (partial) weightbearing is allowed from day 0. In case of revision surgery, poor joint quality, or other complications, the surgeon determines the weightbearing protocol. In multiple-joint procedures involving ankle arthrodesis, 6 weeks of cast immobilization is required, during which no weightbearing was permitted. In case of catheter angiography, this is performed by interventional radiologists prior to TKA to evaluate the presence and extent of synovial hyperemia in the knee. Selective genicular artery embolization is performed in case of synovial hyperemia to reduce hyperemia to limit the chance of postoperative joint bleeding [36]. The life cycle of the prosthesis is monitored biannually after 10 years and annually after 15 years.

### 2.3 Primary outcome

The primary outcome was time to prosthesis failure, measured in years. Prosthesis failure was defined as revision surgery or a documented indication for revision in patients who were ineligible for surgery or declined operative treatment. Time to failure was calculated as the interval between the date of surgery and the date of prosthesis failure. Patients who died during follow-up were censored at the date of death.

### 2.4 Patient characteristics

The following patient characteristics were extracted from the electronic patient records: age at time of surgery (years); type and severity of bleeding disorder; type of surgery (TKA/THA); year of surgery; body mass index (BMI) at time of surgery; treatment regimen at time of surgery (on demand/factor prophylaxis/nonfactor prophylaxis); HIV, hepatitis C, and inhibitor (defined as anti-FVIII above 1.0 or 0.4 for surgeries before and after 2005, respectively) status at time of surgery (positive/negative); multiple joint procedure (yes/no); and degree of arthropathy (defined as total Pettersson score before the first TJR; if unavailable, the score closest to the first TJR date if within 3 years). The Pettersson score rates the 6 joints most prone to HA (elbow, knee, ankle) on a 13-point scale [37]. Higher scores indicate more severe arthropathy.

### 2.5 Perioperative characteristics

The following perioperative data were extracted: difference in pre- and postoperative hemoglobin value (defined as: last known preoperative value minus the first value at day 1 after surgery), length of hospital stay (days), preoperative angiography (yes/no, TKA only), cementation of femoral stem (yes/no, THA only), and postoperative weightbearing protocol (full/limited weightbearing).

### 2.6 Long-term complications

The following data on long-term complications were extracted: postoperative bleeding (yes/no; defined as: hemarthrosis, wound dehiscence, or signs of internal bleeding within 1 month after surgery), PJI (yes/no), and loosening or liner wear diagnosed by the orthopedic surgeon (yes/no) at any time during follow-up.

### 2.7 Analysis

Patient and perioperative characteristics and long-term complications were reported as medians with ranges or frequencies with percentages. Time to prosthesis failure of primary TKA and THA was used as a dependent variable in the Kaplan–Meier survival analyses. PSRs and complication rates were derived in percentages with CIs. Kaplan–Meier results were interpreted until the number of patients at risk (nR) dropped below 10 [38]. Univariable Cox proportional hazards (PH) regression analyses were used to identify predictors for failure. Age, severity of disorder (dichotomized as severe hemophilia A/B or nonsevere and VWD), treatment regimen (dichotomized as on-demand or prophylactic), year of surgery, BMI, HIV status, hepatitis C status, inhibitor status, preoperative angiography (only for TKA), degree of arthropathy, multiple joint procedure, hemoglobin difference, length of hospital stay, preoperative angiography, cementation (only for THA), weightbearing protocol, PJI, and postoperative bleeding were used as independent variables in univariable analysis. Variables with a univariable association *P* < .1 were included in a multivariable model. If multivariable models included postoperative variables, a post hoc analysis including only preoperative variables were conducted to increase the clinical applicability of the results. Fisher’s exact test was used to test differences between complication rates before and after 2000. The year 2000 was chosen as it was used in the most relevant meta-analysis and marked important advances in hemophilia care [8]. Missing data was handled by multiple imputation with predictive mean matching with 5 imputations. Results were pooled afterward. A sensitivity analysis was performed to correct for multiple episodes of TJR in the same individuals at different time points; the same analyses were repeated including only the patient’s first surgery. For patients whose first surgery was bilateral, every right side was included. Significance was set at *P* < .05. All analyses were performed in RStudio (2025) with use of packages “survival,” “mice,” “icenReg,” and “survminer”.

## 3 Results

After data extraction, 128 TKAs and 64 THAs were eligible for inclusion. One patient (with 2 TKAs and 1 THA) was lost to follow-up after being transferred to another hospital in 2018 and was censored at that moment. On average, for TKA, 5% of data was missing across 14 variables, and for THA, 13% was missing across 9 variables. Patient and perioperative characteristics are presented in Table 1.

**Table 1:** Patient and perioperative characteristics.

Characteristic	TKA	THA
Patient characteristics		
Number of joints (*n*)	128	64
Hemophilia		
Severe A	100 (78%)	49 (77%)
Severe B	13 (10%)	6 (9%)
Moderate A	12 (9%)	9 (14%)
VWD	3 (2%)	0 (0%)
Age at time of surgery (y)	51 (26-77)	57 (27-77)
BMI (kg/m^2^)	25.7 (18.3-35.4)^a^*(n = 69)*	24.7 (18.9-42.2)^a^*(n = 41)*
Had surgery after 2000	82 (64%)	45 (70%)
Treatment regimen		
On demand	47 (37%)^a^*(n = 126)*	20 (31%)
Factor prophylaxis	77 (61%)^a^*(n = 126)*	40 (63%)
Emicizumab	2 (6%)^a^*(n = 126)*	4 (6%)
Hepatitis C positive	78 (62%)^a^*(n = 126)*	34 (53%)
HIV positive	12 (10%)^a^*(n = 126)*	3 (5%)
Inhibitor present^b^	3 (3%)^a^*(n = 108)*	3 (5%)^a^*(n = 57)*
Petterson score before first surgery	48 (3-78)	49 (0-78)
Multiple joint procedure		
With ankle arthrodesis	15 (12%)	1 (2%)
Bilateral	24 (19%)	4 (6%)
Bilateral and ankle arthrodesis	12 (9%)	0 (0%)
TKA and THA (unilateral)	6 (5%)	6 (9%)
TKA, THA, and ankle arthrodesis	1 (1%)	1 (2%)
Perioperative characteristics		
Δ Hb (preop vs postop, mmol/L)	2.1 (0.5-5.9)	2.2 (0.7-4.9)
Length of hospital stay (d)	17 (4-148)	12 (7-111)
Preoperative angiography	85 (67%)^a^*(n = 126)*	NA
Cemented femoral stem	NA	26 (43%)^a^*(n = 60)*
Weightbearing protocol		
Full	54 (43%)^a^*(n = 126)*	41 (75%)^a^*(n = 55)*
Partial	43 (34%)^a^*(n = 126)*	13 (24%)^a^*(n = 55)*
No weightbearing	29 (23%)^a^*(n = 126)*	1 (2%)^a^*(n = 55)*

Values are median (range) or frequency (percentage) or as otherwise indicated; percentage might not add up to 100% due to rounding.

Hb, hemoglobin; NA, not applicable; THA, total hip arthroplasty; TKA, total knee arthroplasty; VWD, von Willebrand disease.

a Percentages are not measured from *n* due to missing data.

b Hemophilia A only (*n* = 112 and *n* = 58).

Most patients had severe hemophilia A (TKA 78%, THA 77%) and were treated with factor prophylaxis (TKA, 61%, THA 63%). Median age at surgery was 51 years for TKA and 57 years for THA. Positivity for hepatitis C was present in 62% and 53%, HIV in 10% and 5%, and inhibitors in 3% and 5%, respectively. The degree of arthropathy was already severe before the first surgery, with median Pettersson scores of 48 (TKA) and 49 (THA). In 36% (*n* = 71) of the cases, the surgery was part of a multiple joint procedure, with bilateral procedures (TKA 19%, THA 6%) and combinations with ankle arthrodesis (TKA 12%, THA 2%) performed most often. Half of the patients who underwent TKA were allowed full weightbearing on the prosthesis (43%), although this was more common after THA (75%). In 67% (*n* = 85) of the TKAs, preoperative angiography was performed. The femoral stem was cemented in 40% (*n* = 26) of the THAs.

Postoperative bleeding occurred in 32% of the TKAs (*n* = 40); furthermore, TKA was complicated by both PJI (6%) and loosening (10%). In THAs, postoperative bleeding occurred in 18% (*n* = 10), a PJI was present in 3% of the cases, while loosening occurred in 16%. Complication rates did not differ significantly before and after 2000 (*P* > .05) and are presented in Table 2.

**Table 2:** Long-term complication rates of primary total knee and total hip arthroplasty.

Complication	TKA	THA
	*n* = 128	*n* = 64
Postoperative bleeding	32% (24%-41%)^a^*(n=126)*	18% (9%-30%)^a^*(n = 55)*
Prosthetic joint infection	6% (3%-12%)^a^*(n = 126)*	3% (0.4%-11%)^a^*(n = 61)*
Loosening	10% (6%-17%)^a^*(n=127)*	16% (8%-28%)^a^*(n = 62)*

	nB = 46, nA = 82	nB = 19, nA = 45
Postoperative bleeding		
Before 2000	28% (16%-44%)	38% (10%-51%)
After 2000	33% (23%-44%)	11% (4%-25%)
Fisher’s exact *P* value	.69	.15
Prosthetic joint infection		
Before 2000	11% (4%-24%)	5% (0%-28%)
After 2000	6% (2%-14%)	2% (0%-13%)
Fisher’s exact *P* value	.49	.51
Loosening or liner wear on X-ray		
Before 2000	7% (2%-19%)	26% (10%-51%)
After 2000	12% (6%-22%)	11% (4%-25%)
Fisher’s exact *P* value	.38	.15

Values are proportion (95% CI).

nA, number of patients with surgery after 2000; nB, number of patients with surgery before 2000; THA, total hip arthroplasty; TKA, total knee arthroplasty.

a Percentages are not measured from n due to missing data.

### 3.1 Prothesis survival rates

**Figure 1:**
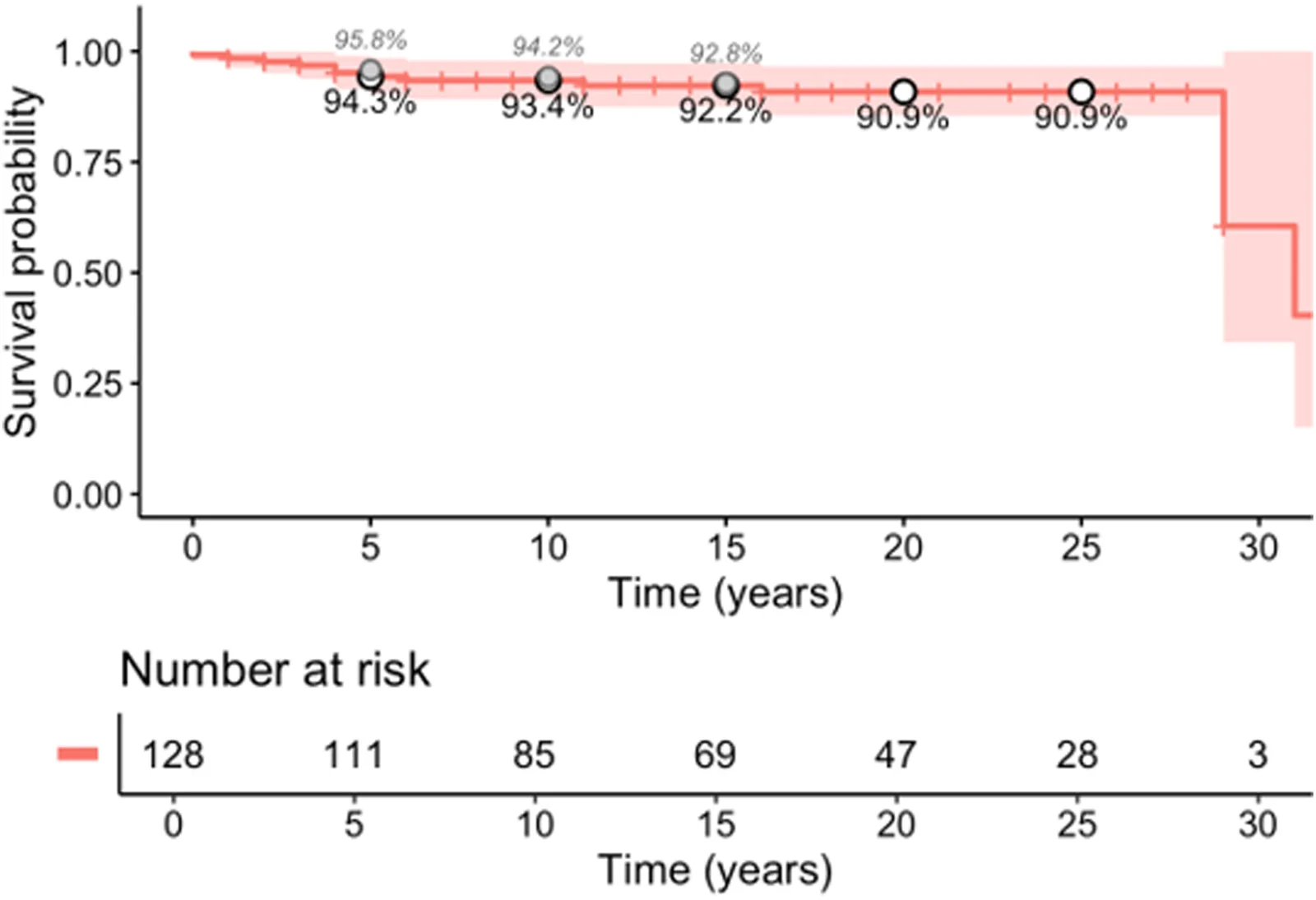
Survival curve for primary total knee arthroplasty in people with bleeding disorders (red line), with 5-, 10-, and 15-year general population survival rates shown as gray dots.

**Figure 2:**
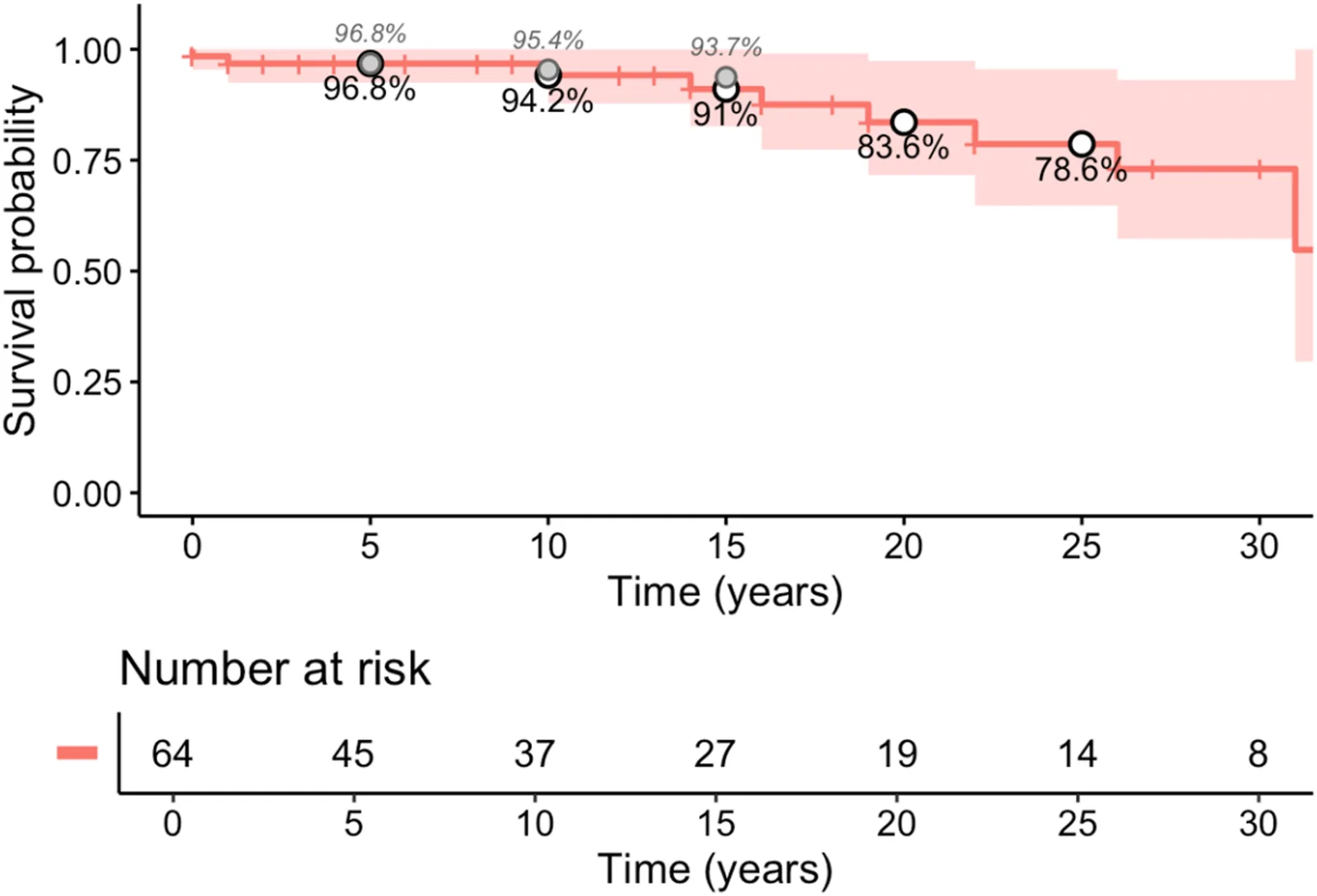
Survival curve for primary total hip arthroplasty in people with bleeding disorders (red line), with 5-, 10-, and 15-year general population survival rates shown as gray dots.

The PSR for TKA in people with bleeding disorders was 92.2% (nR = 71; 95% CI, 87.5-97.3) after 15 years and 90.9% (nR = 28; 95% CI, 85.5-96.6) after 25 years. The PSR for THA in people with bleeding disorders was 91.0% (nR = 27; 95% CI, 82.8-100) after 15 years and 78.6% (nR = 14; 95% CI, 64.8-95.5) after 25 years. The survival curves are presented in Figures 1 and 2. Individual reasons for failure are presented in Table 3.

**Table 3:** Individual failure reasons.

Patient No.	Total knee arthroplasty (n = 128)			Total hip arthroplasty (n = 64)		
	Failure reason	Year of surgery	Years to failure	Failure reason	Year of surgery	Years to failure
1	Liner wear	1990	31	Loosening	1998	31
2	Liner wear	1992	29	Loosening	1992	16
3	Liner wear	1994	29	Liner wear	1993	26
4	Infection	1995	2	Loosening	1995	19
5	Loosening	1996	11	Loosening	2001	14
6	Infection	1996	16	Fracture	2003	22
7	Infection	1999	3	Dislocation	2008	0
8	Disabling functional impairment	2001	4	Loosening	2013	10
9	Infection	2009	4	Infection	2013	1
10	Infection	2012	1			
11	Infection	2017	5			
12	Disabling functional impairment	2017	6			
13	Infection	2017	0			

Among failed TKAs (*n* = 13), early failure (within 5 years, *n* = 8) was caused by PJI in 86% of the cases, while in cases of late failure (after 5 years, *n* = 5), this was only in 17%. Among failed THAs (*n* = 9), early failure was very low (*n* = 2), while almost every late failure (*n* = 7) was due to loosening (*n* = 6, 86%).

### 3.2 Predictors for prosthesis failure

Univariable Cox PH regression analysis for TKA showed that patients with an inhibitor at time of surgery had 16.3 times higher risk for prosthesis failure (*P* = .03) , and patients with PJI at any time during follow-up had 35.9 times higher risk (*P* < .001) (Supplementary Table). A trend was observed such that patients with moderate hemophilia and those with VWD had 4.0 times (*P =* .07) higher risk for prosthesis failure, and patients receiving on-demand treatment had 3.1 times (*P* = .09) higher risk. In the multivariable analysis, PJI (*P* = .03) was the only significant predictor (Table 4). In a post hoc multivariable analysis with only preoperative variables available, inhibitor status (*P* = .08) was the sole variable showing a trend (Table 5). Therefore, PJI is assumed to mediate the association between inhibitor status and PSR; patients with an inhibitor have an increased risk of PJI, and PJI increases the risk of prosthetic failure. In addition, on-demand therapy, nonsevere hemophilia, and inhibitor status are known to be interrelated.

**Table 4:** Multivariable Cox proportional hazards regression: predictors for failure of primary total knee and hip arthroplasty.

Variable	Exponent	95% CI	*P*
Total knee arthroplasty (n = 128)			
Severity of disorder *0 = severe* *1 = nonsevere*	0.28	0.045-1.79	.23
Treatment regimen *0 = on demand* *1 = prophylaxis*	0.65	0.16-2.58	.57
Inhibitor status *0 = absent* *1 = present*	2.90	0.25-33.57	.47
Prosthetic joint infections *0 = no* *1 = yes*	14.39	2.59-79.98	.03^a^
Total hip arthroplasty (n = 64)			
Age	1.06	0.99-1.14	.14
*Continuous*			
Cemented femoral stem *0 = yes* *1 = no*	3.57	0.78-16.67	.16

a Significant at *P* < .05.

**Table 5:** Post hoc multivariable Cox proportional hazards regression: predictors for failure of primary total knee arthroplasty (*n* = 128) (only preoperative variables at baseline).

Variable	Exponent	95% CI	*P*
Severity of disorder	0.39	0.08-1.92	.29
*0 = severe*			
*1 = nonsevere*			
Treatment regimen	0.46	0.13-1.62	.27
*0 = on demand*			
*1 = prophylaxis*			
Inhibitor status	14.83	0.56-141.3	.08
*0 = absent*			
*1 = present*			

Univariable Cox PH regression analysis for THA showed a trend toward higher risk of prosthesis failure in older people than in younger people (*P* = .07; Supplementary Table). Each additional year of age at the time of surgery increased the risk by 1.08. A trend toward higher risk for prosthesis failure was also observed for patients with a cemented femoral stem (*P* = .09). These patients had 4.5 times higher risk than patients with an uncemented femoral stem. In multivariable Cox PH analysis, no significant predictors were observed (Table 4). Age and cementation were moderately correlated (*r* = 0.6), showing that older patients more often received a cemented femoral stem. Because neither variable remained significant in the multivariable model, this may indicate a mediating relationship: age influences the choice for cementation, which is subsequently associated with risk of failure. This is supported by age not being a significant predictor in a subset with only uncemented THA (*P* = .74).

### 3.3 Sensitivity analysis

After selecting every first TJR in the dataset, all patient and perioperative values and long-term complication rates in the sensitivity analysis were within 5% difference of those in the primary analysis, even all PSRs. For both TKA and THA, the same predictors were observed (data not shown).

## 4 Discussion

### 4.1 Summary of findings

The PSR for primary TKA in patients with end-stage HA was 92.2% at 15 years and 90.9% at 25 years. The PSR for primary THA in patients with end-stage HA was 91.0% at 15 years and 78.6% at 25 years. In TKA, PJI during follow-up, which was more prevalent in patients with an inhibitor, was a strong predictor of prosthesis failure. In THA, cementation of the femoral stem, which was associated with a higher age, showed a trend in predicting prosthesis failure.

### 4.2 Comparisons with the literature

The 15-year PSRs for TKA and THA in our cohort of patients with bleeding disorders are similar to those of the general Dutch population (92.8% for TKA and 91.1% for THA) [26] and substantially higher than those previously reported in hemophilia populations (80%-84% for TKA and not available for THA) [8,14]. The hemophilia cohorts in previous studies are similar to the present cohort of people with bleeding disorders in terms of demographics, such as severity and inhibitor status, as well as peri- and postoperative coagulation protocols. Also, bleeding rates were similar, 32% vs 34%. Conversely, in comparison to the general Dutch population, our cohort of people with bleeding disorders consisted of younger, predominantly male patients who more often had multijoint involvement. This highlights the characteristics of HA, which starts at an early age and severely affects multiple joints. Besides demographic differences, infection rates and bleeding rate also differed between our cohort of people with bleeding disorder and the general population: 6% vs 1.2% in TKA and 3% vs 1.5% in THA for infections and 25% vs 3% for bleeding [39,40].

PJI and the presence of an inhibitor were significant predictors for prosthesis failure in TKA. In previous research, these factors were also associated with adverse outcomes. PJI in particular is known as the most frequent complication after TKA and the most common reason for revision [41]. In the general population, PJI is known to be more prevalent in young, male patients with preoperative anemia, prolonged operation duration and hospital stay, postoperative transfusions, and bilateral procedures [41]. All of these are characteristic features of people with bleeding disorders compared with the general population. The effect of having an inhibitor at the time of surgery is described in a few other studies, mostly in case reports, and did not lead to strong evidence or consensus on higher complication rates or shorter survival [42]. Therefore, the significant, higher risk for prosthesis failure in patients with an inhibitor in our study could possibly be explained by these patients having higher risk for PJI through the described risk factors and the higher risk for (subclinical) bleeding.

In THA, age has not been described as a predictor of prosthesis failure in previous hemophilia research. In the general population, a younger age is associated with an increased risk of revision surgery [43]. In the present study, however, a higher risk of prosthesis failure was observed among older patients and patients receiving cemented femoral stems. Recent studies also show improved survival rates in cementless hips, potentially due to improved osteointegration [44]. Cemented stems are more frequently used in older patients and in those with poor bone quality. Moreover, during the study period, cementation of the femoral stem was standard practice for all THA patients aged >65 years. Consequently, the observed association between cemented stems and PSR appears to be mediated by age, as demonstrated in the multivariable analysis. These findings suggest that cementation is a surrogate marker for age and bone quality, while also explaining why older patients have a higher risk for failure in this cohort of people with bleeding disorders: higher age correlates with worse bone quality, especially in individuals with bleeding disorders.

Higher PSRs compared with previous hemophilia populations and the reduction in annual bleeding rate after 2000 may indicate that outcomes improved over time due to advances in hemophilia care. However, year of surgery was not a significant predictor of prosthesis failure in this study, and, in contradiction with Fenelon et al. [8], the present study showed no decrease in long-term complication rates after 2000 compared with before 2000 (*P* > .05). Lastly, recent studies suggest that modern biomaterials, including oxidized zirconium femoral components with highly crosslinked polyethylene used in TKA and ceramic femoral heads in THA, may contribute to improved prosthesis survival. However, these factors were not included in our analyses and therefore could not be assessed in the present study [32,44]. As modern biomaterials were unavailable during the earliest inclusion period, participants enrolled at that time did not receive these prostheses. Consequently, prosthesis survival may improve further with the increasing use of these newer materials.

### 4.3 Strengths and limitations

One strength of the study is that it contains rich, single-center data of high quality due to standardized treatment, thorough registration through the years, and complete follow-up of every patient for lifelong care. Furthermore, the use of a strict inclusion criterion (factor levels <5%) ensured that only patients with defined HA were included. Another strength of this study is that we matched our endpoint (first revision) to the large Dutch Arthroplasty Register (LROI) osteoarthritis database, in combination with a clear separation between end-stage HA patients and osteoarthritis patients in the general population, allowing a strong comparison. Lastly, the high methodological quality of the study is confirmed by the fact that multiple sensitivity analyses gave very similar results. A limitation of our study is the limited statistical power of the multivariable analyses due to the low number of prosthesis failures. In the TKA model, 4 variables were included despite the rule of thumb [45,46] suggesting a maximum of approximately 2 variables. In the THA model, 2 variables were included, whereas the number of events would ideally only support univariable analyses. Nevertheless, the multivariable models provide additional clinical insight, although their results should be considered exploratory, and replication studies with different cohorts are needed to confirm the results. Lastly, although missingness was generally low, this was not the case for the BMI variable. Specifically, BMI data of an earlier date (before 2007) were largely missing due to the transition to electronic patient records (before 2007). As a result, the findings of the corresponding univariable analyses may be biased. However, these results were not statistically significant and do not affect our key message.

### 4.4 Clinical relevance

Given that current PSRs are comparable to those in the general population, TJR in people with bleeding disorders appears more feasible and should be approached with less concern regarding failure. Healthcare professionals can therefore provide more deliberate advice to patients by directly incorporating the identified predictors of failure into their consultation, especially by using results from the post hoc analysis with only preoperative variables. As people with bleeding disorders are typically younger and present with severe joint damage, these PSRs help address the clinical dilemma of whether to delay arthroplasty. Our findings support considering joint replacement at a younger age when clinically indicated. Earlier surgery may in particular be beneficial for THA, as it increases the possibility of using an uncemented femoral stem, and bone quality appears to play an important role in prosthesis survival, given that loosening is the primary cause of failure in THA at longer follow-up. In TKA, additional anti-infectious measures could be used, especially in patients who also have an inhibitor at time of surgery, as early failure is mostly infection-related, and late failure due to loosening is rare in this population.

### 4.5 Recommendations for future research

The present study reports reliable and long-term PSRs for TKA and THA in people with bleeding disorders. However, functional outcomes were not considered. Functional outcomes have been assessed in previous studies and appear promising, although these studies share the same limitations as the PSR literature: long-term data, extending beyond 2 decades and reflecting developments in hemophilia care, are lacking. Therefore, future research should use the data of the present cohort and connect it to functional outcome measures. This would further strengthen TKA and THA as earlier interventions and effective therapeutic options for improving quality of life in patients with end-stage HA.

## 5 Conclusion

In our single-center Dutch sample, the PSR of TKA and THA for people with bleeding disorders with end-stage HA was comparable to that of the general population in literature. Postoperative infection, more common in patients with an inhibitor (TKA) and a cemented femoral stem, associated with a higher age (THA) were predictors for prosthesis failure.

## References

[1] Gualtierotti R., Solimeno L.P., Peyvandi F. (2021). Hemophilic arthropathy: current knowledge and future perspectives. J Thromb Haemost.

[2] Brands M.R., Haverman L., Muis J.J., Driessens M.H.E., van der Meer F.J.M., Goedhart G. (2023). Patients’ and health care providers’ perspectives on quality of hemophilia care in the Netherlands: a questionnaire and interview study. Res Pract Thromb Haemost.

[3] van Leeuwen F.H.P., van Bergen E.D.P., Timmer M.A., van Vulpen L.F.D., Schutgens R.E.G., de Jong P.A. (2023). Magnetic resonance imaging evidence for subclinical joint bleeding in a Dutch population of people with severe hemophilia on prophylaxis. J Thromb Haemost.

[4] Nijdam A., Foppen W., De Kleijn P., Mauser-Bunschoten E.P., Roosendaal G., van Galen K.P. (2016). Discontinuing early prophylaxis in severe haemophilia leads to deterioration of joint status despite low bleeding rates. Thromb Haemost.

[5] Manco-Johnson M.J., Abshire T.C., Shapiro A.D., Riske B., Hacker M.R., Kilcoyne R. (2007). Prophylaxis versus episodic treatment to prevent joint disease in boys with severe hemophilia. N Engl J Med.

[6] Hassan S., van Balen E.C., Smit C., Mauser-Bunschoten E.P., van Vulpen L.F.D., Eikenboom J. (2021). Health and treatment outcomes of patients with hemophilia in the Netherlands, 1972-2019. J Thromb Haemost.

[7] van Heel D.A.M., Foppen W., Fischer K. (2024). Arthropathy on X-rays in 363 persons with hemophilia: long-term development, and impact of birth cohort and inhibitor status. Res Pract Thromb Haemost.

[8] Fenelon C., Murphy E.P., Fahey E.J., Murphy R.P., O’Connell N.M., Queally J.M. (2022). Total knee arthroplasty in hemophilia: survivorship and outcomes-a systematic review and meta-analysis. J Arthroplasty.

[9] Rosas S., Buller L.T., Plate J., Higuera C., Barsoum W.K., Emory C. (2021). Total knee arthroplasty among Medicare beneficiaries with hemophilia A and B is associated with increased complications and higher costs. J Knee Surg.

[10] Kildow B.J., Politzer C.S., DiLallo M., Bolognesi M.P., Seyler T.M. (2018). Short and long-term postoperative complications following total joint arthroplasty in patients with human immunodeficiency virus, hepatitis B, or hepatitis C. J Arthroplasty.

[11] Jones M.D., Buckle C.L. (2020). How does aseptic loosening occur and how can we prevent it?. Orthop Trauma.

[12] Rodriguez-Merchan E.C. (2024). Total hip arthroplasty in hemophilic patients: are their results similar to those of nonhemophilic patients?. Arch Bone Jt Surg.

[13] Rodriguez-Merchan E.C., Mosconi M., De la Corte-Rodriguez H., Jannelli E., Pasta G. (2024). Total knee arthroplasty in people with hemophilia: higher incidence of periprosthetic joint infection and 1-year revision/re-operation than the general population and lower prosthetic survival when early postoperative bleeding complications occurred: current literature review. J Clin Med.

[14] Goddard N.J., Mann H.A., Lee C.A. (2010). Total knee replacement in patients with end-stage haemophilic arthropathy: 25-year results. J Bone Joint Surg Br.

[15] Zingg P.O., Fucentese S.F., Lutz W., Brand B., Mamisch N., Koch P.P. (2012). Haemophilic knee arthropathy: long-term outcome after total knee replacement. Knee Surg Sports Traumatol Arthrosc.

[16] Santos Silva M., Rodrigues-Pinto R., Rodrigues C., Morais S., Costa E., Castro J. (2019). Long-term results of total knee arthroplasty in hemophilic arthropathy. J Orthop Surg (Hong Kong).

[17] Oyarzun A., Barrientos C., Barahona M., Martinez A., Soto-Arellano V., Courtin C. (2020). Knee haemophilic arthropathy care in Chile: midterm outcomes and complications after total knee arthroplasty. Haemophilia.

[18] Li Z., Feng B., Du Y., Wang Y., Bian Y., Weng X. (2020). Complications of total knee arthroplasty in patients with haemophilia compared with osteoarthritis and rheumatoid arthritis: a 20-year single-surgeon cohort. Haemophilia.

[19] Anazor F.C., Uthraraj N., Relwani J. (2023). Postoperative outcomes of total elbow replacement in haemophilic elbow arthropathy: a systematic review. Haemophilia.

[20] Carulli C., Innocenti M., Tambasco R., Perrone A., Civinini R. (2023). Total knee arthroplasty in haemophilia: long-term results and survival rate of a modern knee implant with an oxidized zirconium femoral component. J Clin Med.

[21] Challoumas D., Munn D., Jeyakumar G., Bagot C., Rodgers R., Kearns R. (2024). Outcomes of total hip and knee arthroplasty in patients with haemophilia: a meta-analysis of comparative studies and clinical practice recommendations. Haemophilia.

[22] Ernstbrunner L., Hingsammer A., Imam M.A., Sutter R., Brand B., Meyer D.C. (2018). Long-term results of total elbow arthroplasty in patients with hemophilia. J Shoulder Elbow Surg.

[23] Gillinov S.M., Burroughs P.J., Moore H.G., Rubin L.E., Frumberg D.B., Grauer J.N. (2022). Total hip arthroplasty in patients with classic hemophilia: a matched comparison of 90-day outcomes and 5-year implant survival. J Arthroplasty.

[24] Goker B., Caglar O., Kinikli G.I., Aksu S., Tokgozoglu A.M., Atilla B. (2022). Postoperative bleeding adversely affects total knee arthroplasty outcomes in hemophilia. Knee.

[25] Davey M.S., Hurley E.T., Gaafar M., Molony D., Mullett H., Pauzenberger L. (2021). Long-term outcomes of total elbow arthroplasty: a systematic review of studies at 10-year follow-up. J Shoulder Elbow Surg.

[26] Landelijke Registratie Orthopedische Implantaten/Dutch Arthroplasty Register (LROI) (2024). Annual Report 2024. https://www.lroi.nl/media/prqogokg/pdf-lroi-report-2024.pdf.

[27] Rodriguez-Merchan E.C., De la Corte-Rodriguez H., Alvarez-Roman T., Gomez-Cardero P., Encinas-Ullan C.A., Jimenez-Yuste V. (2022). Complications and implant survival of total knee arthroplasty in people with hemophilia. J Clin Med.

[28] Saris D.B., van Rinsum A.C., Dhert W.J., Roosendaal G., Mali W.P. (1997). Periarticular aneurysm formation in haemophilia. Lancet.

[29] Carulli C., Rizzo A.R., Innocenti M. (2017). Hip arthropathy in haemophilia. J Clin Med.

[30] Chen L., Lin S., Zhou W., Ling Y., Shi Z., Ge Q. (2025). Prosthesis survival situation and complications following total hip arthroplasty in hemophilic patients: a systematic review. BMC Musculoskelet Disord.

[31] Lee S.H., Rhyu K.H., Cho Y.J., Yoo M.C., Chun Y.S. (2015). Cementless total hip arthroplasty for haemophilic arthropathy: follow-up result of more than 10 years. Haemophilia.

[32] Civinini R., Matassi F., Carulli C., Sirleo L., Lepri A.C., Innocenti M. (2017). Clinical results of oxidized zirconium femoral component in tka. a review of long-term survival: review article. HSS J.

[33] Carulli C., Innocenti M., Linari S., Morfini M., Castaman G., Innocenti M. (2021). Joint replacement for the management of haemophilic arthropathy in patients with inhibitors: a long-term experience at a single haemophilia centre. Haemophilia.

[34] Civinini R., Carulli C., Matassi F., Lepri A.C., Sirleo L., Innocenti M. (2017). The survival of total knee arthroplasty: current data from registries on tribology: review article. HSS J.

[35] van Galen K.P., Mauser-Bunschoten E.P., Leebeek F.W. (2012). Hemophilic arthropathy in patients with von Willebrand disease. Blood Rev.

[36] Foppen W., van der Schaaf I.C., van Leeuwen F.H.P., Verlind D.H., van Vulpen L.F.D., Vogely H.C. (2023). Pre-operative synovial hyperaemia in haemophilia patients undergoing total knee replacement and the effects of genicular artery embolization: a retrospective cohort study. Haemophilia.

[37] Pettersson H., Ahlberg A., Nilsson I.M. (1980). A radiologic classification of hemophilic arthropathy. Clin Orthop Relat Res.

[38] Kattan M.W., Vickers A.J. (2020). Statistical analysis and reporting guidelines for CHEST. Chest.

[39] van Veghel M.H.W., Belt M., Spekenbrink-Spooren A., Kuijpers M.F.L., van der Kooi T.I.I., Schreurs B.W. (2024). Validation of the incidence of reported periprosthetic joint infections in total hip and knee arthroplasty in the Dutch Arthroplasty Register. J Arthroplasty.

[40] Smeets M.J.R., Gademan M.G.J., Nelissen R., Cannegieter S.C., Nemeth B. (2025). Age- and sex-specific risks of major cardiovascular complications and all-cause mortality following elective hip and knee arthroplasty in the Netherlands: a Dutch hospital data registry study. Arthroplast Today.

[41] Jeys L.M., Patel R., Jenkins T., Baydar S., Pagkalos J. (2025). The infected total knee replacement: the worst and unfortunately most frequent complication. Orthop Trauma.

[42] Danielson H., Lassila R., Ylinen P., Yrjönen T. (2017). Total joint replacement in inhibitor-positive haemophilia: long-term outcome analysis in fifteen patients. World J Orthop.

[43] Bayliss L.E., Culliford D., Monk A.P., Glyn-Jones S., Prieto-Alhambra D., Judge A. (2017). The effect of patient age at intervention on risk of implant revision after total replacement of the hip or knee: a population-based cohort study. Lancet.

[44] Carulli C., Felici I., Martini C., Civinini R., Linari S., Castaman G. (2015). Total hip arthroplasty in haemophilic patients with modern cementless implants. J Arthroplasty.

[45] Vittinghoff E., McCulloch C.E. (2007). Relaxing the rule of ten events per variable in logistic and Cox regression. Am J Epidemiol.

[46] van Domburg R., Hoeks S., Kardys I., Lenzen M., Boersma E. (2014). Tools and techniques--statistics: how many variables are allowed in the logistic and Cox regression models?. EuroIntervention.

