# Supplementary material for: Long-term prosthesis survival of total hip and total knee arthroplasty in people with inherited bleeding disorders
Source: Res Pract Thromb Haemost. 2026 Jul 28;10(5):106887. doi: 10.1016/j.rpth.2026.106887 (PMC13499158; doi:10.1016/j.rpth.2026.106887)
Supplement: Supplementary file 1 [file mmc1.docx]

| **Supplemental table 1.** Univariable Cox Proportional Hazards regression: time to failure | | | | | | | |
| --- | --- | --- | --- | --- | --- | --- | --- |
|  | **Total Knee Arthroplasty** | | | **Total Hip Arthroplasty** | | | |
|  | HR | CI 95% | p-value | HR | CI 95% | p-value | |
| **Patient** | **n=128** | | | **n=64** | | | |
| **Severity of disorder**  *0 = other*  *1 = severe* | 0.25 | 0.065-0.95 | 0.071 | 0.70 | 0.09-5.88 | 0.752 | |
| **Age at time of surgery**  *Continuous* | 0.96 | 0.90-1.02 | 0.259 | 1.08 | 1.00-1.17 | 0.071 | |
| **Year of surgery**  *Continuous* | 1.02 | 0.95-1.10 | 0.478 | 1.04 | 0.95-1.15 | 0.440 | |
| **BMI**  *Continuous* | 1.06 | 0.90-1.25 | 0.487 | 1.16 | 0.89-1.51 | 0.369 | |
| **Treatment regimen**  *0 = on demand*  *1 = prophylaxis* | 0.32 | 0.10-1.01 | 0.089 | 1.35 | 0.27-6.69 | 0.730 | |
| **Hepatitis C status**  *0 = negative*  *1 = positive* | 0.48 | 0.16-1.44 | 0.211 | 0.76 | 0.17-3.38 | 0.719 | |
| **HIV Status**  *0 = negative*  *1 = positive* | <0.01 | 0-∞ | 0.999 | 2.34 | 0.28-19.33 | 0.462 | |
| **Inhibitor status**  *0 = absent*  *1 = present* | 16.27 | 2.26-117.13 | 0.034* | <0.01 | 0-∞ | 0.999 | |
| **Pettersson score**  *Continuous* | 0.87 | 0.83-0.91 | 0.546 | 0.99 | 0.62-1.58 | 0.961 | |
| **Multiple joint procedure**  *0 = no*  *1 = yes* | 0.74 | 0.21-2.64 | 0.649 | 0.33 | 0.04-2.73 | 0.337 | |
| **Clinical** | **n=128** | | | **n=64** | | | |
| **Hb difference**  *Continuous* | 0.94 | 0.55-1.60 | 0.825 | 1.14 | 0.48-2.69 | 0.774 |  |
| **Length of hospital stay**  *Continuous* | 0.98 | 0.94-1.02 | 0.490 | 1.02 | 1.00-1.04 | 0.122 | |
| **Pre-op angiography**  *0 = no*  *1 = yes* | 3.03 | 0.55-16.73 | 0.246 | NA | NA | NA | |
| **Cemented femoral stem**  *0 = no*  *1 = yes* | NA | NA | NA | 4.55 | 1.03-20 | 0.091 | |
| **Weightbearing protocol**  *0 = full*  *1 = limited* | 2.44 | 0.50-11.82 | 0.307 | 0.97 | 0.18-5.27 | 0.973 | |
| **Follow-up** | **n=128** | | | **n=64** | | | |
| **Post-operative bleeding**  *0 = no*  *1 = yes* | 1.60 | 0.47-5.48 | 0.465 | 0.49 | 0.09-2.55 | 0.973 | |
| **PJI**  *0 = no*  *1 = yes* | 35.87 | 8.9-144.65 | <0.001* | <0.01 | 0-∞ | 0.999 | |
| HR: Hazard Ratio; CI: Confidence Interval; NA; Not Applicable; HIV: Human Immunodeficiency Virus; Hb: Hemoglobin; PJI: Prosthetic Joint Infection; *Significant at p <0.05; ∞ Infinite; | | | | | | | |

ix. **Appendix**
